# Revealing Long-Range Order in Brush-like Graft Copolymers Through In Situ Measurements of X-Ray Scattering During Deformation

**DOI:** 10.3390/polym16233309

**Published:** 2024-11-27

**Authors:** Akmal Z. Umarov, Evgeniia A. Nikitina, Alexey A. Piryazev, Ioannis Moutsios, Martin Rosenthal, Andrey O. Kurbatov, Yulia D. Gordievskaya, Elena Yu. Kramarenko, Erfan Dashtimoghadam, Mitchell R. Maw, Sergei S. Sheiko, Dimitri A. Ivanov

**Affiliations:** 1 Department of Chemistry, Lomonosov Moscow State University (MSU), GSP-1, 1-3 Leninskiye Gory, 119991 Moscow, Russia; umarovakmalum@gmail.com (A.Z.U.); evgeniya1484@gmail.com (E.A.N.); stunnn@gmail.com (A.A.P.); kurbatov@polly.phys.msu.ru (A.O.K.); yulia.gordievskaya@uni-potsdam.de (Y.D.G.); kram@polly.phys.msu.ru (E.Y.K.); 2 Institut de Sciences des Matériaux de Mulhouse-IS2M, CNRS UMR 7361, F-68057 Mulhouse, France; imoutsios@uoi.gr; 3 Department of Chemistry, KU Leuven, Celestijnenlaan 200F, Box 2404, B-3001 Leuven, Belgium; martin.rosenthal@esrf.fr; 4 Department of Chemistry, University of North Carolina, Chapel Hill, NC 27599-3290, USA; edashtimoghadam@troy.edu (E.D.); mitchellmaw1@gmail.com (M.R.M.); sergei@email.unc.edu (S.S.S.)

**Keywords:** tissue-mimetic, brush-like copolymer networks, synchrotron X-ray scattering, uniaxial stretching, dissipative particle dynamics

## Abstract

Brush-like graft copolymers (A-g-B), in which linear A-blocks are randomly grafted onto the backbone of a brush-like B-block, exhibit intense strain-stiffening and high mechanical strength on par with load-bearing biological tissues such as skin and blood vessels. To elucidate molecular mechanisms underlying this tissue-mimetic behavior, in situ synchrotron X-ray scattering was measured during uniaxial stretching of bottlebrush- and comb-like graft copolymers with varying densities of poly(dimethyl siloxane) and poly(isobutylene) side chains. In an undeformed state, these copolymers revealed a single interference peak corresponding to the average spacing between the domains of linear A-blocks arranged in a disordered, liquid-like configuration. Under uniaxial stretching, the emergence of a distinct four-spot pattern in the small-angle region indicated the development of long-range order within the material. According to the affine deformation of a cubic lattice, the four-spot pattern’s interference maxima correspond to 110 reflections upon stretching along the [111] axis of the body-centered unit cell. The experimental findings were corroborated by computer simulations of dissipative particle dynamics that confirmed the formation of a *bcc* domain structure.

## 1. Introduction

Brush-like polymers represent an interesting class of materials characterized by a long backbone with densely grafted side chains. Recent studies have highlighted their unique properties, including biomimetic mechanical behavior, adjustable thermal stability, and the potential for biodegradability through the selection of appropriate side chains [1,2,3,4,5,6,7,8]. By fine-tuning the brush architecture, such polymers can achieve a remarkable combination of ultra-softness and strain-adaptive stiffening (or firmness) [9,10,11], closely mimicking the deformation response of soft biological tissues [12,13,14,15]. Specifically, they have been shown to exhibit intense stiffening upon stretching, a hallmark of many biological tissues [16,17]. Such behavior is unattainable with traditional linear polymers due to chain flexibility and entanglements. Moreover, these materials have shown the potential to enhance the mechanical resilience of biological substrates and support dynamic self-healing processes in tissues [18,19,20,21,22].

The combination of softness and firmness in bottlebrush polymers arises from the low entanglement density of their backbones and the inherent pre-stretching of these backbones, even in an undeformed state [23], where side chains simultaneously act as “solvents” and mediators of backbone rigidity [24]. By adjusting the length of the side chains, it is possible to precisely control the material’s firmness while maintaining a low Young’s modulus [24]. Unlike linear polymers, the backbones of bottlebrush polymers remain pre-stretched without the need for macroscopic deformation [24].

The tissue-mimetic performance was enhanced by implementing linear-bottlebrush-linear ABA triblock copolymer that microphase separate to form physical networks, where bottlebrush strands (B-blocks) are linked by spherical domains of A-blocks) [25,26]. Despite demonstrating tissue-like softness and strain-induced stiffening, these systems exhibited relatively low mechanical strength, markedly weaker than the load-bearing tissues such as skin and blood vessels. The primary cause of this low strength in linear triblock ABA plastomers can be attributed to the “one strand—one molecule” principle, where each polymer backbone functions as a single mechanically active strand. To overcome this limitation, an alternative molecular architecture known as A-g-B was introduced, in which linear A-blocks are randomly grafted along the backbone of the brush-like B-block. In this design, each backbone connects multiple network cells, significantly enhancing the mechanical strength [27]. Furthermore, selective-solvent annealing of these systems can substantially increase the strain-stiffening parameter (β) by promoting network reorganization [28].

Traditional methods to improve molecular packing of block copolymers include surface nanopatterning combined with thermal, vapor, and selective-solvent annealing techniques [29,30,31,32]. Structural evolution during the deformation of microphase-separated morphologies can be monitored using a combination of small-angle X-ray scattering (SAXS) and specialized in situ stages applying oscillatory shear, as well as uniaxial and equibiaxial stretching [33,34,35]. In these studies, supramolecular ordering typically manifests as a cubic *bcc* lattice of spherical microdomains. In situ mechanical experiments provide insights into the nature of deformation (affine versus non-affine) and the transformation of dispersed phase structures, often transitioning from spherical to prolate spheroidal shapes [36]. Alternatively, the evolution of the structure factor during stretching can be examined for nanocomposites through in situ Atomic Force Microscopy (AFM) [37].

To gain a deeper understanding of the biomimetic mechanical behavior of brush-like graft copolymers, a comprehensive investigation was conducted employing synchrotron X-ray scattering in combination with in situ uniaxial mechanical stretching. This study systematically examined a series of bottlebrush and comb-like graft copolymers with varying grafting densities of poly(dimethyl siloxane) and poly(isobutylene) side chains, intercalated with linear graft chains of poly(methyl methacrylate) and polystyrene. The real-time X-ray scattering patterns were analyzed using an affine deformation model and subsequently compared with computer simulations based on the dissipative particle dynamics method. This combined experimental and computational effort elucidates the structural transformations in bottlebrush physical networks during mechanical deformation and, for the first time, reveals the hidden order of the microphase-separated domain within a brush-like polymer matrix.

## 2. Materials and Methods

**Scheme 1:**
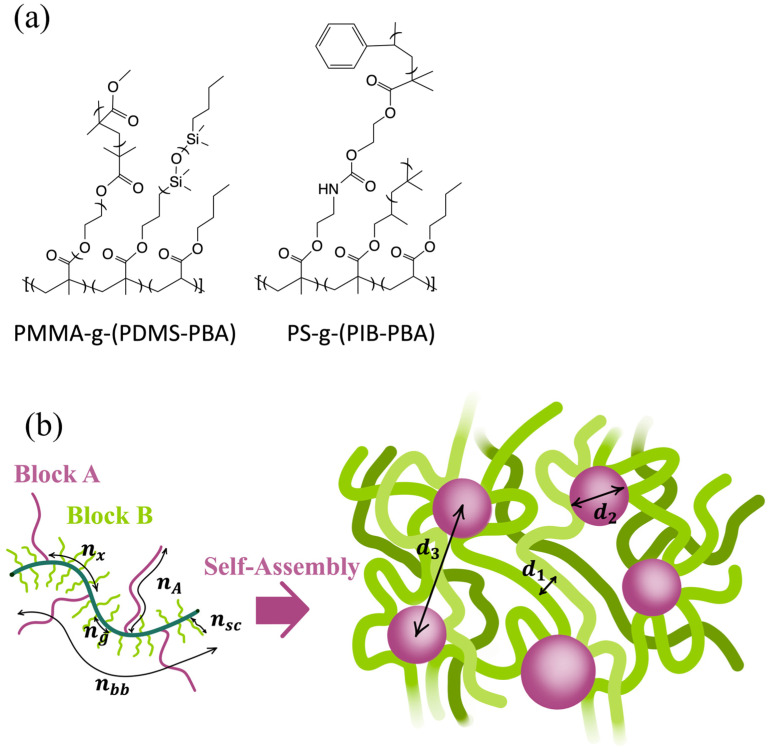
(**a**) Chemical structures of the synthesized **A**-g-**B** graft copolymers where **A** stands for poly(methyl methacrylate) or polystyrene and **B** denotes poly(dimethylsiloxane) or poly(isobutylene). (**b**) Self-assembly of the **A**-g-**B** graft copolymers in a physical network composed of nanometer-sized domains of **A** connected by the bottlebrush blocks. The variable structural parameters include the length of the bottlebrush backbone n_BB_, length of side chains n_sc_, length of the graft block n_A_, and distance between the grafted blocks n_x_. Upon self-assembly, the system forms domains of block **A** with a diameter d_2_ separated by a distance d_3_ and a diameter of the **B**-block equal d_1_.

Three series of BB elastomers with two different types of graft blocks (polymethylmethacrylate and polystyrene) and two different types of side chains (poly(isobutylene) and polydimethylsiloxane)) were synthesized. The synthetic procedures used are described elsewhere [27,28,38]. The chemical structures of the synthesized copolymers are shown in Scheme 1a. The degrees of polymerization (DP) of side chains ($n_{sc}$), the DP of the backbone between crosslinks ($n_{x}$), DP and volume fraction of the **A**-block, DP of total brush backbone, and number of spacer repeat units between side chains are provided in Table 1. The microstructural parameters of the self-assembled structure of the copolymers are specified in Scheme 1b.

### 2.1. X-Ray Scattering

The small-angle X-ray scattering (SAXS) experiments were carried out at the BM26 and ID02 beamlines of the European Synchrotron Radiation Facility (ESRF) in Grenoble (France). At the BM26 beamline, measurements were conducted in transmission geometry using 13 keV photon energy, covering a q range from 8.0 × 10^−2^ nm^−1^ to 4.0 nm^−1^ (|**q**| = 4π sin(θ)/λ, where θ is the Bragg angle and λ is the wavelength). A Pilatus 1M detector, with an active area of 169 mm × 179 mm, recorded SAXS intensity at a 2.8 m sample-to-detector distance. At the ID02 beamline, measurements were performed with 12.2 keV photon energy using an Eiger2 4M detector placed 2.8 m from the sample, covering a q range from 0.03 to 2.5 nm^−1^.

The data correction, calibration, and integration were performed using the fast azimuthal integration Python library [39].

In situ tensile experiments were carried out using a Linkam MFS350 stretching device with a 20 N force-beam module installed. Dogbone-shaped samples were gently fixed in the clamps and preliminarily covered with fine sandpaper to prevent sliding of the samples during stretching.

The diameter of the **A**-block domains was determined from the SAXS curves by fitting them to the form factor of spheres.

(1) $$P\left(q\right)=3\left[sin\left(qR\right)-qRcos\left(qR\right)\right]/{\left(qR\right)}^{3}$$

The analytical expression in Equation (1) was convoluted with a Gaussian distribution to account for the scattering from a polydisperse population of spheres.

### 2.2. Computer Simulations

Further insights into the microstructure and strain response of brush-like graft copolymer melts have been provided by computer simulations that corroborate the experimental results.

#### 2.2.1. DPD Model

Molecular dynamics simulations of an **A**-g-**B** copolymer melt have been performed using the dissipative particle dynamics (DPD) method [40]. In our modeling, a graft copolymer is constructed from a backbone chain of $n_{bb}$ DPD beads, each connected to either a short side chain of $n_{sc}$ beads or a longer one of $n_{A}$ beads. Interactions between beads are characterized by a pairwise conservative force $F_{ij}^{C}$, a bond stretching force $F_{ij}^{B}$ (for bonded beads), a dissipative force $F_{ij}^{D}$, and a random force $F_{ij}^{R}$. The total force acting on the i-th bead is given by the following:

(2) $$F_{i}=\sum_{j\neq i}\left(F_{ij}^{C}+F_{ij}^{B}+F_{ij}^{D}+F_{ij}^{R}\right)$$

The soft-core repulsion between i-th and j-th beads is implemented by the following:

(3) $$F_{ij}^{C}=\left\{\begin{array}{c} a_{\alpha \beta }\left(1-\frac{r_{\mathrm{ij}}}{R_{c}}\right)r_{ij}/r_{\mathrm{ij}},\;r_{\mathrm{ij}}\leq R_{c}\\ 0,r_{\mathrm{ij}}>R_{c} \end{array}\right.$$

where $r_{ij}$ is the vector between i-th and j-th bead, $a_{\alpha \beta }$ is the repulsion parameter between α-type and β-type beads, and $R_{c}$ is the cutoff distance, which is taken as the length scale, $R_{c}=1.0$. We took the conservative DPD parameter between similar beads $a_{ii}=150.0$ and used the empirical relation $\chi_{\alpha \beta }\approx 0.3∆a_{\alpha \beta }=0.3(a_{\alpha \beta }-a_{ii}$) obtained in the work [41] to evaluate other repulsion parameters, where the incompatibility between species is characterized by the Flory–Huggins parameter $\chi_{\alpha \beta }$. Following the experimental systems, we assumed that beads of short sidechains are compatible with backbone units, while long sidechains are incompatible with both backbone chains and short sidechains. For compatible beads, we used $\chi_{\alpha \beta }=0$, for incompatible $\chi_{\alpha \beta }=\chi$.

The connectivity between individual beads is modeled via a simple spring force:

(4) $$F_{ij}^{B}=-K(r_{\mathrm{ij}}-l_{0})r_{ij}/r_{\mathrm{ij}}$$

where K=150.0 is the bond stiffness and $l_{0}$ = 0.5 is the equilibrium bond length. These selected parameters, together with $a_{ii}=150.0$ allow us to move on to non-phantom polymers [42]. Other two contributions, a dissipative force $F_{ij}^{D}$ and a random force $F_{ij}^{R}$, are expressed by the following:

(5) $$F_{ij}^{D}=-\gamma (1-r_{\mathrm{ij}}/R_{c}{)}^{2}(r_{ij}\cdot v_{ij})r_{ij}/r_{\mathrm{ij}}^{2}$$

(6) $$F_{ij}^{R}=\sigma (1-r_{\mathrm{ij}}/R_{c})\xi_{ij}\Delta t^{-1/2}r_{ij}/r_{\mathrm{ij}}$$

where $v_{ij}=v_{i}-v_{j}$ is the relative velocity, $\xi_{ij}$ is a random number with zero mean and unit variance, Δt is the timestep, and γ and σ are parameters defining the magnitude of the dissipative and random forces, correspondingly. In our simulations, we used σ=3, γ=4.5, and the integration timestep of Δt=0.02. The graft copolymer melt was modeled in a cubic simulation box of the size $40^{3}$, containing about $1.92\times 10^{5}$ DPD beads. The value of the Flory–Huggins parameter was increased from χ=0 to χ=100 in a stepwise protocol. At each intermediate χ-value, the system was equilibrated from a few tens to several hundred million steps.

#### 2.2.2. Mechanical Response

The mechanical properties of the graft copolymers were studied under uniaxial stretching along the x-axis while keeping the system volume constant. In order to calculate the stress–strain curves, the system was subjected to a tensile strain in the x-direction, whereby its initial linear size ($L_{0}$) was extended to a specified final size (L). The ratio of these two sizes ($\lambda =L/L_{0}$) represents the strain ratio. The system was deformed at a constant strain rate in such a way that a twofold strain of the sample was achieved in 30 million steps. The stress tensor was averaged for $10^{5}$ steps during the strain and calculated using the virial theorem as follows:

(7) $$p_{\alpha \beta }=\frac{1}{V}\sum_{i}v_{i\alpha }v_{i\beta }+\frac{1}{V}\sum_{i}\sum_{j>i}\left(F_{i\alpha }^{C}+F_{i\alpha }^{B}\right)r_{ij\beta }$$

where indices α,β=x,y,z, V is the volume of the system, $v_{i}$ is the velocity of the *i*th particle, and $r_{ij}$ is the distance between i and j particles. The true stress was calculated as follows:

(8) $$\sigma_{true}=-\left\langle p_{xx}\right\rangle +\left\langle \frac{p_{yy}+p_{zz}}{2}\right\rangle$$

We performed at least 4 runs starting from different initial states and averaged the data to obtain the final stress–strain curve.

The calculated stress–strain curve was fitted to the equation of state, which describes $\sigma_{true}\left(\lambda \right)$ dependence [43] as

(9) $$\sigma_{true}=\frac{E_{0}}{3}\left(\lambda^{2}-\lambda^{-1}\right)\left[1+2{\left(1-\frac{\beta \left(\lambda^{2}+2\lambda^{-1}\right)}{3}\right)}^{-2}\right]/\left(1+\frac{2}{{\left(1-\beta \right)}^{2}}\right)$$

where $E_{0}$ is the Young’s modulus and $\beta =\langle R^{2}\rangle /\langle R_{max}^{2}\rangle$ defines the extensibility of network strands relative to their contour length $\langle R_{max}^{2}\rangle$.

#### 2.2.3. Calculation of Scattering

To elucidate the microstructure of the graft copolymer melt and its evolution with strain, the scattering patterns of the initial equilibrated state of the system, as well as several intermediate states along the stress–strain curve, were calculated. For each state, the scattering intensity was averaged over 200 snapshots, with an interval of $10^{5}$ DPD steps between them. Some calculations were performed analogously to the experiment:

(10) $$I\left(q\right)=\frac{1}{n}\left\langle {\left|\sum_{j=1}^{n}e^{iqr_{j}}\right|}^{2}\right\rangle$$

where n is the total number of beads. The averaging was performed over the scattering vector set {$q_{r}$, $q_{\varphi }\}$, where $q_{r}$ has 2000 points within the range [0.05∗2π/σ,0.095∗2π/σ] and $q_{\varphi }$ has 180 points within the range [0,2π]. These calculations are important to show the scattering pattern along and perpendicular to the stretching direction. To show the structure factor of the system in the initial underformed state, it is useful to consider all directions over the scattering vector set {$q_{x}$, $q_{y},q_{z}\}=\{2\pi k/l_{x},2\pi k/l_{y},2\pi k/l_{z},\}$, where k, m, and p are integers from 1 to 32 and over a sequence of 800 independent system conformations.

## 3. Results

### 3.1. Sample Structure in the Initial Undeformed State

The microstructure of the brush-like graft copolymers in their undeformed state was investigated using synchrotron small-angle X-ray scattering (SAXS). Key structural parameters obtained from the SAXS profiles include the position of the characteristic “bottlebrush peak” at $q_{1}^{*}$ [44,45,46,47], the diameter of the **A**-block spheres d_2_, and the inter-sphere distance d_3_. The aggregation number Q, which represents the number of **A**-blocks within a single sphere, was calculated as follows:

(11) $$Q=\frac{\pi d_{2}^{3}}{6n_{A}\nu_{A}}$$

where $n_{A}$ denotes the degree of polymerization (DP) of the **A**-block and $\nu_{A}$ stands for the monomer volume of the **A**-block. The surface area per individual brush-like block at the **A**-**B** interface was determined using the following expression:

(12) $$S_{in}=\frac{\pi d_{2}^{2}}{2Q}$$

In Equation (12), we account for the fact that each **A**-block is connected to two **B**-strands. The surface area per bottlebrush block in the bulk (S_0_) was calculated as follows:

(13) $$S_{0}=\frac{2d_{1}^{2}}{\sqrt{3}}$$

Equation (13) assumes that the backbones of the comb or bottlebrush copolymers are arranged in a 2D hexagonal lattice, where d_1_ represents the d-spacing of the (10) diffraction peak of this lattice. The resulting microstructural parameters of the studied copolymers are summarized in Table 2.

All studied materials demonstrate exceptional softness, with elastic modulus values ranging between 20 and 60 kPa. The copolymer structure consists of spherical domains formed by the linear graft **A**-block, which are embedded within a continuous matrix of the **B**-block. The interdomain distance (d_3_) depends on both the length of the **A**-block (n_A_) and the spacing between adjacent blocks along the main backbone (n_x_). Although the domain size (d_2_) generally increases with n_A_, it exhibits significantly smaller values compared to those observed in previously studied linear **ABA** triblock copolymers [46].

The position and intensity of the bottlebrush peak are primarily governed by the grafting density of the side chains (n_g_). For densely grafted brushes, the peak is sharp and well defined, but as the n_g_-values increase, the peak progressively broadens and diminishes in intensity. At sufficiently high n_g_-values, the peak completely disappears, aligning with the structural characteristics of linear polymers.

The organization of the brush blocks at the **A**/**B** phase interface is quantified by the interface area per block (S_in_) compared to the bulk structure (S_o_). In all systems studied, S_in_ is significantly smaller than S_o_, indicating that the brush blocks are severely constrained at the interface.

### 3.2. Evolution of the Sample Structure in the Course of Uniaxial Deformation

**Figure 1:**
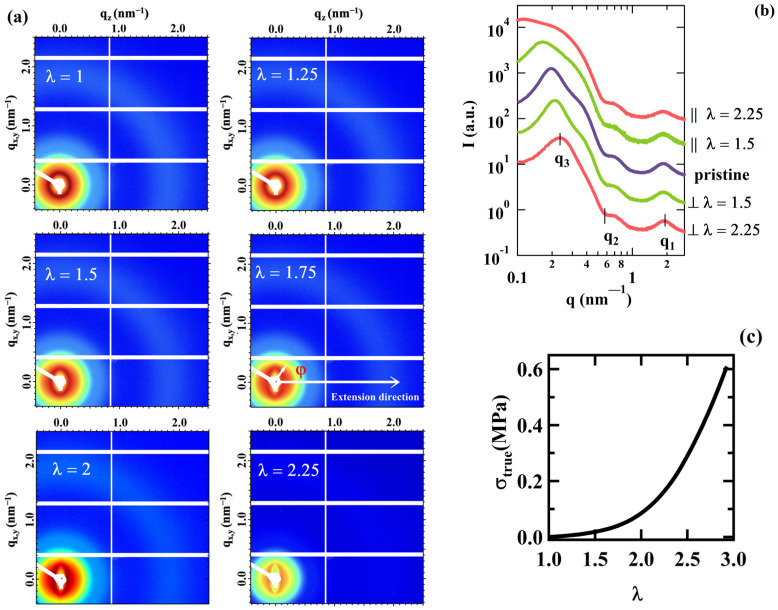
(**a**) Representative 2D scattering patterns obtained during uniaxial stretching of sample PDMS-PMMA_3. The extension direction is parallel to the z-axis. (**b**) Corresponding 1D diffraction profiles extracted from the patterns in (**a**), along the directions parallel and perpendicular to the stretching axis. The characteristic distances, including interdomain spacing (d_3_), form factor of the **A**-block domains (d_2_), and bottlebrush peak (d_1_), are indicated. The λ values are specified for each profile. (**c**) Stress–elongation curve for the same sample.

**Figure 2:**
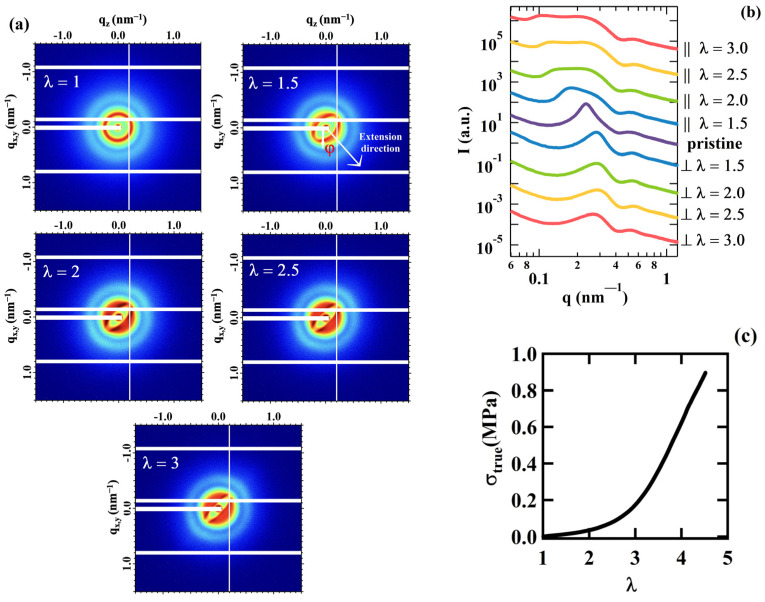
(**a**) Representative 2D scattering patterns obtained during uniaxial stretching of sample PIB_PS_2. The extension direction is oriented at a 45° angle relative to the horizontal axis. (**b**) Corresponding 1D diffraction profiles extracted from the patterns in (**a**), along the directions parallel and perpendicular to the stretching axis. The λ values are specified for each profile. (**c**) Stress–elongation curve for the same sample.

Figure 1 and Figure 2 display the X-ray scattering results obtained during uniaxial stretching of selected bottlebrush and comb-like graft copolymers alongside their respective stress–elongation curves. Here, λ represents the dimensionless elongation ratio. In the undeformed state (λ = 1), both samples exhibit X-ray patterns with a prominent isotropic SAXS peak, corresponding to the interdomain distance, d_3_. This peak, being the only feature in the small-angle region, indicates that the **A**-domains are arranged in short-range liquid-like order. Upon stretching, the initially circular SAXS peak transforms into an elliptical shape, with the short axis aligned parallel to the stretching direction. This elliptical distortion corresponds to an increase in the distance between **A**-domains along the strain direction.

One-dimensional sections of the 2D patterns illustrate the evolution of scattering intensity along both parallel and perpendicular directions relative to the stretching axis (Figure 1b and Figure 2b). The observed shift of the primary interference maximum to lower q values contrasts with the perpendicular direction, where an opposite trend is observed. Notably, the interference maximum broadens substantially along the stretching direction, whereas the form factor of the domains remains nearly unchanged.

**Figure 3:**
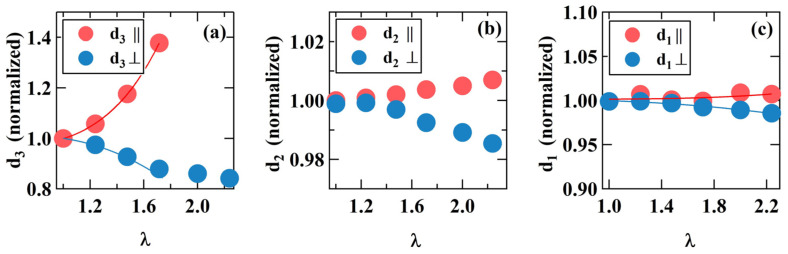
(**a**–**c**) Variation of the normalized values of d_1_, d_2,_ and d_3_, respectively, as a function of λ during the stretching of sample PDMS-PMMA_3. The values of d_3,‖_ are not measurable above λ of 1.75. The solid lines in (**a**) represent fits based on Equation (14).

**Figure 4:**
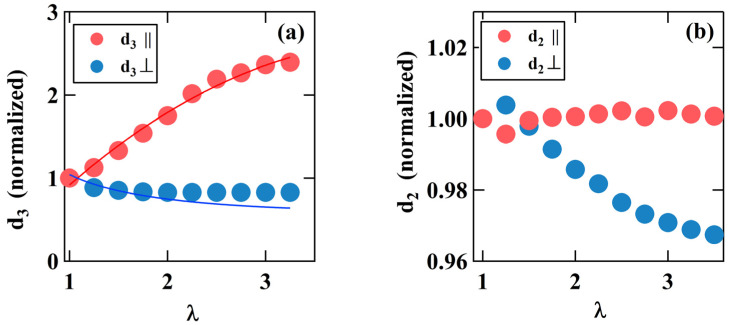
(**a**,**b**) Variation of the normalized values of d_3_ and d_2_, respectively, as a function of λ during the stretching of sample PIB_PS_2. The solid lines in (**a**) represent fits based on Equation (14).

The evolution of the normalized d_3_-spacing as a function of λ is shown in Figure 3a and Figure 4a. The normalized d-spacing is calculated using the formula: $d_{normalized}\left(\lambda \right)=d\left(\lambda \right)/d\left(\lambda =1\right)$. The solid lines represent the relationship between the d_3_ values in the directions parallel and perpendicular to the stretching direction (d_3,‖_ and d_3,⊥_), following the equation:

(14) $$d_{3,\|}d_{3,⊥}^{2}=const$$

Equation (14) assumes a constant volume for the specimen. During stretching, uniaxial elongation of the sample along the drawing direction ($d_{3,\|}$) is expected to be accompanied by isotropic contraction ($d_{3,⊥}$) in the plane perpendicular to the drawing axis. It can be seen that despite a somewhat nonlinear variation of d_3_ with λ, the relation (14) is valid until λ of 1.75 for sample PDMS-PMMA_3 and λ of approximately 2.5 for sample PIB_PS_2. For sample PDMS-PMMA_3, this relation cannot be confirmed in the full range of available extensions, as the primary interference maximum along the stretching direction becomes indistinct.

The size of the **A**-block domains exhibits minimal variation during extension (see Figure 3b and Figure 4b), likely due to the glassy nature of the **A**-block phase. Despite this limited change, clear indications suggest that the domain size in the direction perpendicular to the stretching shows a slightly decreasing trend, which overall may imply a reduction in domain volume—and, consequently, a decrease in the aggregation number—with stretching.

**Figure 5:**
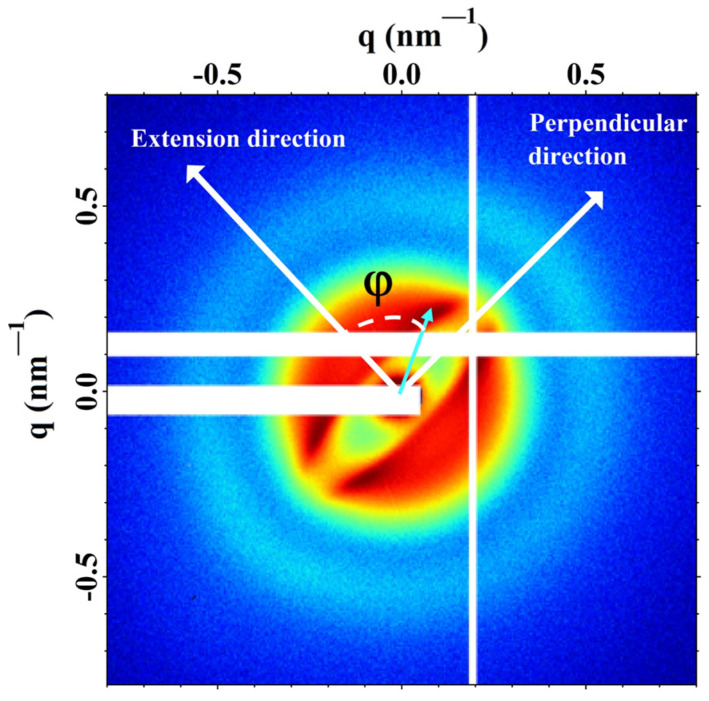
Detailed view of the small-angle region: the distinctive four-spot pattern characteristic of the deformed brush copolymers. The azimuthal angle (φ) is defined as the angle between the direction of the SAXS maxima and the axis of stretching.

As far as the organization of the bottlebrushes is concerned, sample PDMS-PMMA_3 shows that upon stretching, the distance between the backbones in the direction perpendicular to stretching decreases, which is in line with the telescoping deformation model reported previously [48]. Most notably, deformation leads to a dramatic change in the isotropic intensity of the d_3_ peak. Instead of the initially isotropic azimuthal intensity distribution, a four-spot pattern emerges (Figure 5), suggesting that the deformed sample exhibits a series of reticular planes, with their normal inclined at an angle φ relative to the stretching direction. The results of in situ stretching experiments on the additional bottlebrush and comb-like copolymers listed in Table 1 are presented in the Supporting Information (Figures S1–S6). This observation contrasts with the initial liquid-like organization, where long-range order is not apparent.

### 3.3. Analysis of the Structural Evolution Using Analogy with Cubic Lattices

**Figure 6:**
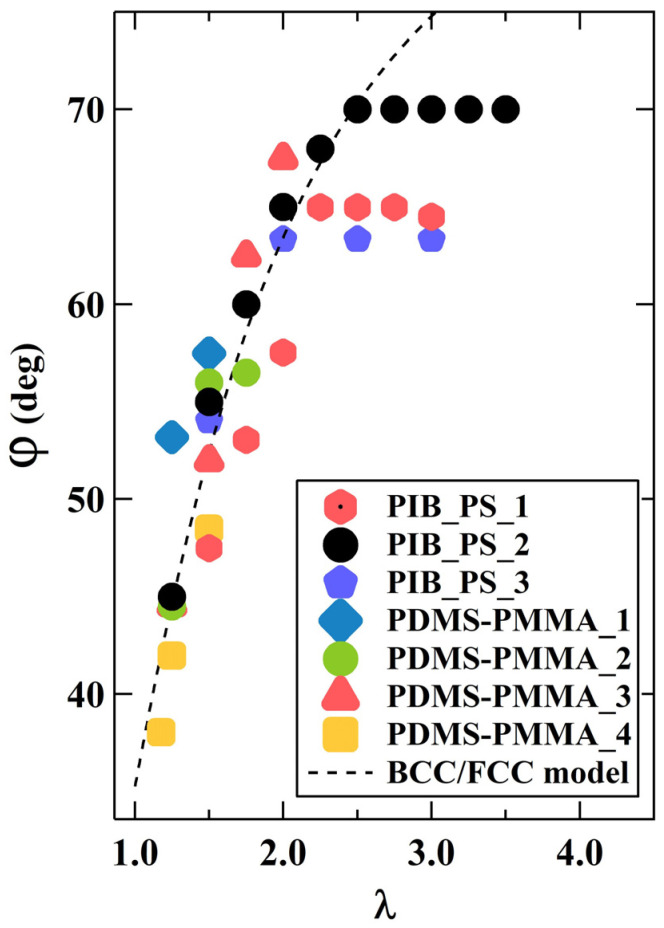
Variation of the azimuthal angle (φ) as a function of the drawing ratio (λ) for the analyzed comb- and brush-like copolymers. The dashed line represents the analytical prediction based on Equation (15), illustrating the angle between the [111] direction and the normal to the (110) planes of a bcc lattice under the assumption of affine deformation.

To gain further insight into the structural evolution during uniaxial stretching, we plotted the angle φ—defined as the angle between the stretching direction and the SAXS maxima associated with the four-spot pattern—as a function of λ (Figure 6). The data reveal that as λ increases, φ increases from approximately 35° to around 70°. Notably, data points from all samples align along a single master curve, indicating a consistent structural evolution across samples. This observation suggests that the initial liquid-like state may possess an underlying, latent, long-range order.

**Figure 7:**
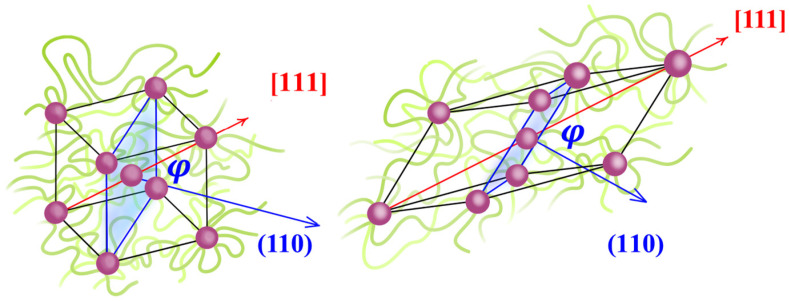
Schematic representation of the affine stretching of a *bcc* lattice along the [111] direction, illustrating the rotation of (110) planes induced by the applied deformation.

Similar four-spot SAXS patterns have previously been observed during in situ stretching of sphere-forming block copolymers, including polystyrene-block-poly(ethylene-co-butylene)-block-polystyrene triblock copolymer [34] and blends of polymethylmethacrylate-block-poly(n-butyl-acrylate) diblock copolymer with polymethylmethacrylate-block-poly(n-butylacrylate)-block-polymethylmethacrylate triblock copolymer [49]. In contrast to the copolymers examined in this study, these previously investigated samples displayed long-range order in their undeformed state, typically organized in a cubic *bcc* lattice. To facilitate a quantitative comparison of the deformation behavior of our materials with that of *bcc*-type block copolymers, we consider the affine stretching of a *bcc* lattice along the [111] direction in Figure 7, where the stretching direction represents the shortest path connecting the lattice nodes.

During deformation, the change in angle between the stretching direction and the normal to the (110) plane can be monitored. This plane produces the strongest diffraction peak in the lattice, appearing at the lowest q-value. The schematic in Figure 7 illustrates that this angle increases with λ, consistent with our experimental observations above. The analytical expression for the angle between the [111] direction and the normal to the (110) plane can be derived from the equations of the respective planes as a function of the sample elongation λ:

(15) $$\varphi =arccos\left(\sqrt{\frac{2}{2+\lambda^{3}}}\right)$$

The analytical dependence is represented by the dashed line in Figure 6, showing reasonable agreement with the experimental data points.

At higher deformations, certain samples demonstrate systematic deviations from the behavior expected for cubic structures. For instance, in sample PIB_PS_2, the $\varphi \left(\lambda \right)$ relationship appears to level off around *λ* = 2.5. This point aligns with the onset of pronounced deformation stiffening, as shown in Figure 2c, suggesting a possible correlation between structural reorganization and mechanical response at this stage of stretching. Similar observations can be made for samples PIB_PS1 and PIB_PS_3 (Figure S7). Additional insights into the structural organization of the graft copolymers under study can be obtained through computer simulations.

It is noteworthy that an *fcc* lattice, where the stretching axis aligns along [110], and the first diffraction peak corresponds to (111), would yield an identical curve. Thus, although the behavior of the samples closely parallels that of cubic structures, it remains challenging to distinguish definitively between the two cubic lattice types in this analysis.

### 3.4. Modeling of the Sample Structure in the Undeformed State and During Uniaxial Deformation

DPD simulations of brush-like graft copolymer melt allowed us to study in detail its structural features in the initial undeformed state and its structural evolution under uniaxial deformation. The parameters of the coarse-grained model of an **A**-g-**B** brush-like graft copolymer were chosen as follows: the number of DPD beads in the backbone, **B** side chains, and **A** grafts are equal to n_bb_ = 51, n_sc_ = 3, and n_A_ = 5, respectively. The copolymer contains three **A** grafts, regularly distributed along the backbone, so that the number of **B** side chains in each subchain between **A**-blocks along the backbone is equal to 16 (the terminal segments contain eight short side chains each). All DPD beads of the graft copolymer are assumed to be of the same size, combining several (6–10) monomer units of PIB/PDMS and PS/PMMA blocks; thus, the bead size σ in the simulation corresponds to approximately 21.5 Å in the real systems.

For simplicity, the backbone and the side chains are assumed to be of the same chemical nature (**B**-type). Strong segregation of the **A**-blocks within the **B** matrix is modeled by the high value of the Flory–Huggins parameter χ_AB_ ≡ χ = 100. Note that according to our coarse-graining approach, each interacting bead can be mapped onto a segment of the side chains, which consists of several monomer units, so that the χ parameter describes these coarse-grained interactions. At the resulting high value of the incompatibility parameter 3χ_AB_ n_A_ = 1500, the morphology of the aggregates should not change for a given composition [50].

**Figure 8:**
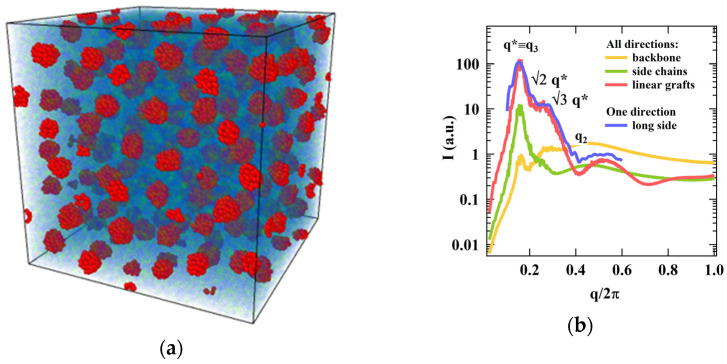
(**a**) Snapshot of the system at *λ* = 1, suggesting a structural arrangement closely resembling a *bcc* lattice. The backbone and side chain beads are shown in 90% transparent colors. (**b**) The structure factor for various components within the bottlebrush melt, as well as the scattering intensity from the hydrophobic beads along a single direction, derived from simulation experiments.

Strong incompatibility of the **A** grafts and bottlebrush side chains induces microphase separation within the melt, resulting in the formation of spherical aggregates by the **A**-blocks (Figure 8a). For the selected system parameters, the size distribution of these **A**-block domains approximates a normal distribution (Figure S8) centered around a mean size of 14. The slight rightward shift in the distribution suggests the presence of a limited number of larger aggregates within the system.

The structure factor S(q) of the **A**-g-**B** melt exhibits three distinct maxima, which suggest a *bcc* arrangement of spherical **A**-domains in the bottlebrush matrix (Figure 8b). The first peak at q* corresponds to the q_3_ peak observed in the experimental data. The calculated *d*-spacing d_3_ is 9.33σ = 20 nm, which aligns well with the experimental range for interdomain distances. The domain size d_2_, derived from the form factor minimum position at q_2_, is 1.7σ = 3.65 nm. The values extracted from the scattering data closely match those calculated from the size distributions. The observed discrepancy in domain sizes between simulation and experimental results can be attributed to the relatively shorter **A**-block length used in the simulations.

**Figure 9:**
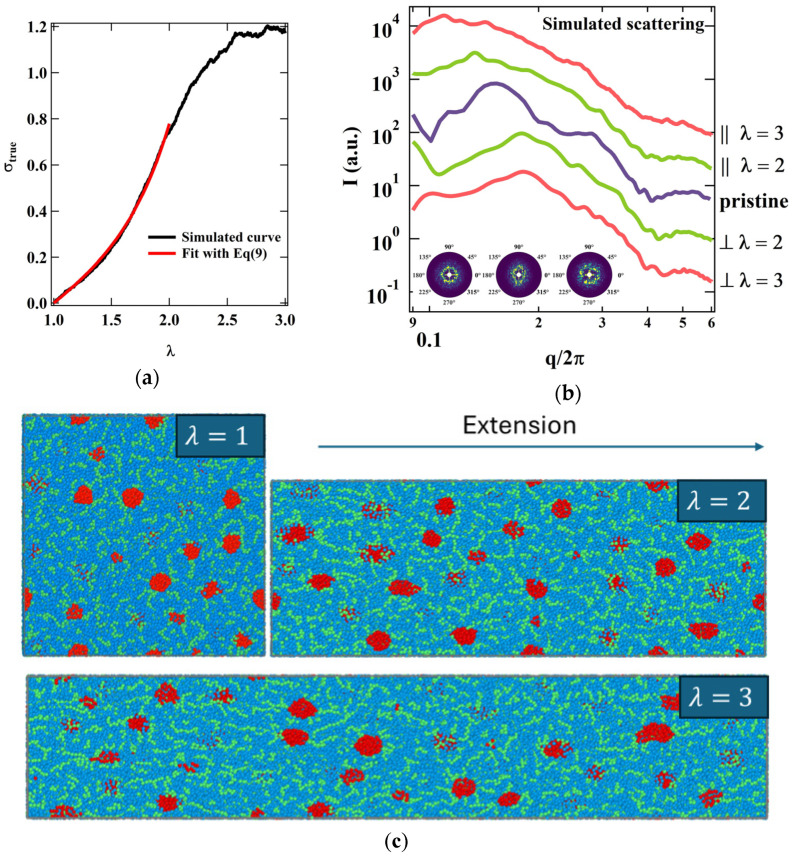
(**a**) The true stress dependence on *λ* = *L*/*L*_0. The black line corresponds to simulation results, and the red line corresponds to fits with Equation (9). (**b**) Scattering intensity profiles calculated for the hydrophobic beads along the stretching direction and perpendicular to it, with corresponding 2D SAXS patterns shown as insets. (**c**) Snapshots of the system at different drawing ratios *λ*.

In the graft copolymer, multiple segregating **A**-blocks facilitate microphase separation, resulting in the formation of a physical network where brush-like strands interconnect, and the **A**-block aggregates serve as network nodes. The fraction of elastically active strands, derived from the bimodal distribution of the end-to-end distance calculated for backbone subchains between **A**-block grafting points, is 0.74. The network’s response to uniaxial tensile strain (Figure 9a) exhibits strong non-linearity, with pronounced strain-stiffening attributed to the finite extensibility of the brush-like strands. A strain-stiffening parameter of β = 0.27, determined by fitting the stress–strain curve to the equation of state (refer to Section 2.2.2), is lower than the experimental β ≈ 0.4-0.7 determined for the n_g_=1 systems yet closely agrees with n_g_ = 4 and n_g_ = 8 systems (cf. Table 1 and [27]). This is logical, as each bead in the simulation represents several monomeric units. At elongation ratios exceeding 2, the sample begins to yield.

Figure 9c presents snapshots of the system at different deformation stages. The **A**-domains show slight elongation along the stretching direction, accompanied by a change in the domain size distribution and an increase in the interdomain distance. Scattering patterns recorded along and perpendicular to the strain (Figure 9b) corroborate these observations. The domain structure shows only minor modifications throughout the deformation. At λ = 2, the average aggregation number increases to 14.5, and at λ = 3, it reaches 15.5, as shown in the size distributions (Figure S8). With stretching, the distribution broadens, and its peak shifts slightly rightward. Notably, new aggregates begin to form at λ = 2, reflected by the increase in the right shoulder of the distribution. By λ = 3, partial dissociation of **A**-domains starts, leading to an increase in the left shoulder, indicating the formation of smaller clusters.

## 4. Discussion

The structural evolution of brush-like copolymers under mechanical stretching, as corroborated by coarse-grained simulations, implies a higher internal order in these systems than initially suggested by SAXS measurements on undeformed samples. The scattering profiles reveal only a single pronounced interference peak at small angles, which is insufficient for definitive structural assignment to a specific lattice. It is possible that additional annealing steps could be necessary to equilibrate the structure, as has been observed in other sphere-forming block copolymers where annealing effectively enhances structural organization, reflected in more definitive SAXS patterns [34]. The nonequilibrium state of the brush copolymer melt is further indicated by the reduced surface area occupied by the copolymer backbones compared to the same values observed in the melt (cf. Table 2). Thus, mechanical stress may be required to reveal the internal order, potentially promoting the arrangement of **A**-domains via repulsive interactions. These interactions are mediated by the interfacial zones surrounding the domains, where the brushes remain in a constrained configuration.

Comparing the microstructural and mechanical parameters of the brush copolymer melts obtained through experiments and simulations provides valuable insights. For the first time, simulations using the DPD technique have been performed to evaluate the structure factors and examine the effects of uniaxial deformation on the structural organization of the brush-like copolymers. The simulations accurately replicate the deformation-induced stiffening, closely matching experimental observations for bottlebrush and comb-like copolymers (see Figure 1c and Figure 2c, and Figure S7 in the Supporting Information). Experimentally, this steep upturn in the stress–elongation curve coincides with a reduction in the **A**-domain volume and a leveling-off of the $\varphi \left(\lambda \right)$ dependence, indicating a deviation from affine deformation behavior [49]. In previous studies on a series of polystyrene-block-poly(ethylene-co-butylene)-block-polystyrene and polystyrene-block-polyisobutylene-block-polystyrene triblock copolymers, deformation of glassy PS domains within the soft PIB matrix was observed under strain [36]. The authors attributed this effect to the high concentration of stress within the PS domains during deformation. In our research, the deformation of PMMA domains in the PDMS-PMMA graft copolymers during stretching was first reported in [27]. Therefore, it seems plausible that as λ increases, the deformation of the glassy **A**-domains reaches a point where **A**-segments begin to detach, resulting in a reduction in the aggregation number. In the simulations, the **A**-domain size distribution varies significantly more upon stretching (Figure S8). This can be explained by the fact that the **A**-domains remain mobile in the simulations, whereas in the room-temperature experiments, they are in a glassy state.

## 5. Conclusions

The deformation-induced structural evolution was investigated for a series of brush-like graft copolymers (**A**-g-**B**), where linear **A**-blocks are randomly grafted onto a brush-like **B**-block backbone to demonstrate exceptional tissue-mimetic softness and firmness, characterized by Young’s modulus ranging between 20 and 60 kPa and up to β = 0.46, respectively. The copolymer structure consists of spherical domains formed by the linear graft **A**-block, which are embedded within a continuous matrix of the **B**-block. In their initial, undeformed state, these copolymers exhibit a single interference peak in small-angle X-ray scattering, reflecting the average distance between **A**-domains arranged in a disordered form. As uniaxial stretching progresses, distinct four-spot patterns appear in the small-angle scattering region, indicating the emergence of long-range order. The evolution of the pattern with increased strain aligns with theoretical expectations for affine deformation in a cubic lattice. Coarse-grained simulations further validated the formation of a body-centered cubic (*bcc*) domain structure, supporting the experimental findings. The simulations accurately replicate the deformation-induced stiffening in the stress–elongation curves of bottlebrush copolymers, observed at extensions around 1.5, and yield a precise estimate of the stiffening parameter (β) of 0.27. These findings underscore the structural transformations triggered by stretching, which play a key role in imparting biomimetic mechanical properties to the copolymers. The developed approach is well suited for investigating the emergence of long-range order in a variety of sphere-forming block copolymers. However, its applicability is limited by transitions from cubic structures to alternative phase-separated morphologies, such as cylindrical structures, as the volume fraction of the dispersed phase increases.

## Figures and Tables

**Table 1:** Molecular characteristics of the synthesized brush-like **A**-g-**B** graft copolymers.

Sample Name	n_sc_ ^1^	n_x_ ^2^	n_A_ ^3^	Φ_A_ ^4^	n_BB_ ^5^	n_g_ ^6^	E_0_ ^7^ (kPa)	λ_max_ ^8^	β ^9^
poly[MMA-g-(PDMS/PMMA)]									
PDMS-PMMA_1	14	149	53	0.029	210	1	16.9	1.92	0.42
PDMS-PMMA_2	14	149	63	0.034	607	1	26.6	2.14	0.42
PDMS-PMMA_3	14	149	62	0.034	1935	1	31.4	2.92	0.40
PDMS-PMMA_4	14	149	81	0.044	1935	1	53.1	2.26	0.46
poly[nBA-ran-MMA-g-(PIB/PS)]									
PIB_PS_1	18	452	60	0.05	1265	8	59.9	3.85	0.16
PIB_PS_2	18	803	120	0.05	N/A	8	25.6	4.75	0.16
PIB_PS_3	18	360	60	0.05	1259	4	51.0	3.63	0.22

^1^ Degree of polymerization (DP) of side chains; ^2^ number average DP of brush backbone between **A**-blocks; ^3^ DP of **A**-blocks; ^4^ volume fraction of **A**-blocks; ^5^ DP of total backbone in the **A**-g-**B** macromolecule; ^6^ number of spacer repeat units between side chains; ^7^ Young’s modulus determined either as the tangent of a stress–strain curve at λ→1 or from the fitting equation $\sigma_{true}\left(\lambda \right)=\frac{G}{3}\left(\lambda^{2}-\lambda^{-1}\right)\left(1+2{\left(1-\frac{\beta \left(\lambda^{2}+2\lambda^{-1}\right)}{3}\right)}^{-2}\right)$ at λ = 1 as $E_{0}=E(1+2{\left(1-\beta \right)}^{-2})/3$; ^8^ elongation at break; ^9^ strain-stiffening parameter obtained by fitting the true stress–strain curves using the equation $\sigma_{true}\left(\lambda \right)$.

**Table 2:** Microstructural parameters of the synthesized brush-like **A**-g-**B** graft copolymers.

Sample Name	d_3_, nm ^1^	d_2_, nm ^2^	d_1_, nm ^3^	Q ^4^	S_in_, nm ^2,5^	S_0_, nm ^2,6^
poly[nBA-ran-MMA-g-(PIB/PS)]						
PIB_PS_1	19.7	11.2	N/A	72	2.7	N/A
PIB_PS_2	27.3	16.8	6.7	121	3.7	51.8
PIB_PS_3	18.4	11.9	N/A	86	2.6	N/A
poly[MMA-g-(PDMS/PMMA)]						
PDMS-PMMA_1	28.5	11.6	3.4	107	2.0	13.3
PDMS-PMMA_2	31.1	14.5	3.4	175	1.9	13.3
PDMS-PMMA_3	32.0	14.9	3.4	193	1.8	13.3
PDMS-PMMA_4	27.6	13.6	3.4	125	2.5	13.3

^1^ Distance between the **A**-block spheres calculated from the *d*-spacing of the main interference peak at q_3_; ^2^ diameter of the **A**-block spheres determined from the SAXS curves by fitting them to the form factor of spheres with a minimum at q_2_; ^3^ *D*-spacing of the bottlebrush peak (q_1_); ^4^ aggregation number corresponding to the **A**-block spheres estimated from diameters of the **A**-block spheres using the corresponding monomer volumes: $\nu_{PMMA}=0.14\;{nm}^{3}$ and $\nu_{PS}=0.17\;{nm}^{3}$; ^5^ surface per each **B**-block at the **A**-**B** interface calculated from d_2_ and Q values; ^6^ surface per each **B**-block in the bulk calculated from the *d*-spacing of the bottlebrush peak.

## Data Availability

Data are contained within the article.

## Supplementary Materials

The following supporting information can be downloaded at https://www.mdpi.com/article/10.3390/polym16233309/s1; X-ray scattering combined with in situ stretching data for samples PDMS_PMMA_1, PDMS_PMMA_2, PDMS_PMMA_4, PIB_PS_1, and PIB_PS_3; stress–elongation curves for PDMS_PMMA bottlebrush copolymers and PIB_PS comb-like copolymers; distributions of aggregation numbers for clusters formed by hydrophobic graft chains at drawing ratios (*λ*) of 1, 2, and 3 extracted from computer simulations. Figure S1: Representative 2D SAXS pattern for sample PDMS_PMMA_1 at λ of 1.25, with the extension directioniented along the z-axis. The left panel shows the four-spot pattern with angle φ indicating the orientation relative to the extension direction. The right panel offers an enlarged view of the central region, illustrating the four-spot pattern characteristic of the sample under deformation; Figure S2: Representative 2D SAXS pattern for sample PDMS_PMMA_2 at λ of 1.5, with the extension direction aligned parallel to the z-axis. The right panel provides a close-up of the central region, highlighting the distinct four-spot pattern; Figure S3: Representative 2D SAXS pattern for sample PDMS_PMMA_4 at λ of 1.5, with the extension direction aligned parallel to the z-axis. The right panel shows the central region, highlighting the distinct four-spot pattern; Figure S4: Selected 1D diffraction profiles extracted from 2D SAXS patterns recorded during in-situ stretching experiments on samples PDMS_PMMA_1 (left), PDMS_PMMA_2 (middle), and PDMS_PMMA_4 (right). Profiles are shown for directions both parallel and perpendicular to the stretching direction, with corresponding drawing ratios indicated; Figure S5: Representative 2D SAXS patterns for samples PIB_PS_1 at λ of 2 (left), and PIB_PS_3 at λ of 1.5 (right), showing the emergence of the characteristic four-spot pattern; Figure S6: Selected 1D diffraction profiles extracted from 2D SAXS patterns recorded during in-situ stretching experiments on samples PIB_PS_1 (left), PIB_PS_3 (right). Profiles are shown for directions both parallel and perpendicular to the stretching direction, with corresponding drawing ratios indicated; Figure S7: Stress-elongation curves for PDMS_PMMA bottlebrush copolymers (left) and PIB_PS comb-like copolymers (right); Figure S8: Distributions of aggregation numbers (M) for clusters formed by hydrophobic graft chains at drawing ratios (*λ*) of 1, 2, and 3, illustrating the evolution of aggregate sizes with increasing deformation. Probability (P) is normalized to unity for each distribution.
