# Supplementary material for: Revealing Long-Range Order in Brush-like Graft Copolymers Through In Situ Measurements of X-Ray Scattering During Deformation
Source: Polymers (Basel). 2024 Nov 27;16(23):3309. doi: 10.3390/polym16233309 (PMC11644117; doi:10.3390/polym16233309)
Supplement: Supplementary file 1 [file polymers-16-03309-s001.zip › polymers-3311168-supplementary.pdf]

Supporting Information for

Article

Revealing Long-Range Order in Brush-like Graft Copolymers Through In Situ Measurements of X-Ray Scattering During Deformation

Akmal Z. Umarov [1], Evgeniia A. Nikitina [1], Alexey A. Piryazev [1], Ioannis Moutsios [2], Martin Rosenthal [3], Andrey O. Kurbatov [1], Yulia D. Gordievskaya [1], Elena Yu. Kramarenko [1], Erfan Dashtimoghadam [4], Mitchell R. Maw [4], Sergei S. Sheiko [4] and Dimitri A. Ivanov [1,2,*]

[1] Department of Chemistry, Lomonosov Moscow State University (MSU), GSP-1, 1-3 Leninskiye Gory, 119991 Moscow, Russia; umarovakmalum@gmail.com (A.Z.U.); evgeniya1484@gmail.com (E.A.N.); stunnn@gmail.com (A.A.P.); kurbatov@polly.phys.msu.ru (A.O.K.); yulia.gordievskaya@uni-potsdam.de (Y.D.G.); kram@polly.phys.msu.ru (E.Y.K.)

[2] Institut de Sciences des Matériaux de Mulhouse-IS2M, CNRS UMR 7361, F-68057 Mulhouse, France; imoutsios@uoi.gr

[3] Department of Chemistry, KU Leuven, Celestijnenlaan 200F, Box 2404, B-3001 Leuven, Belgium; martin.rosenthal@esrf.fr

[4] Department of Chemistry, University of North Carolina, Chapel Hill, NC 27599-3290, USA; edashtimoghadam@troy.edu (E.D.); mitchellmaw1@gmail.com (M.R.M.); sergei@email.unc.edu (S.S.S.)

* Correspondence: dimitri.ivanov@uha.fr

1. X-ray scattering combined with in-situ stretching

1.1. Poly[MMA-g-(PDMS/PMMA)] bottlebrush copolymers

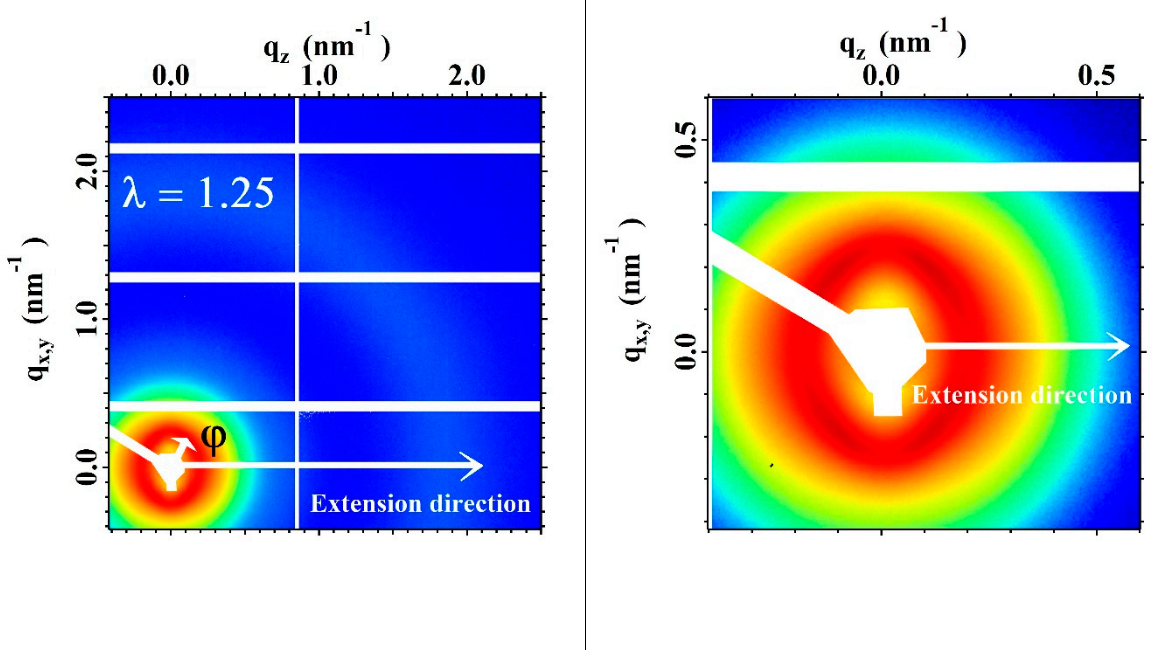

Figure S1. Representative 2D SAXS pattern for sample PDMS_PMMA_1 at λ of 1.25, with the extension direction oriented along the z-axis. The left panel shows the four-spot pattern with angle φ indicating the orientation relative to the extension direction. The right panel offers an enlarged view of the central region, illustrating the four-spot pattern characteristic of the sample under deformation.

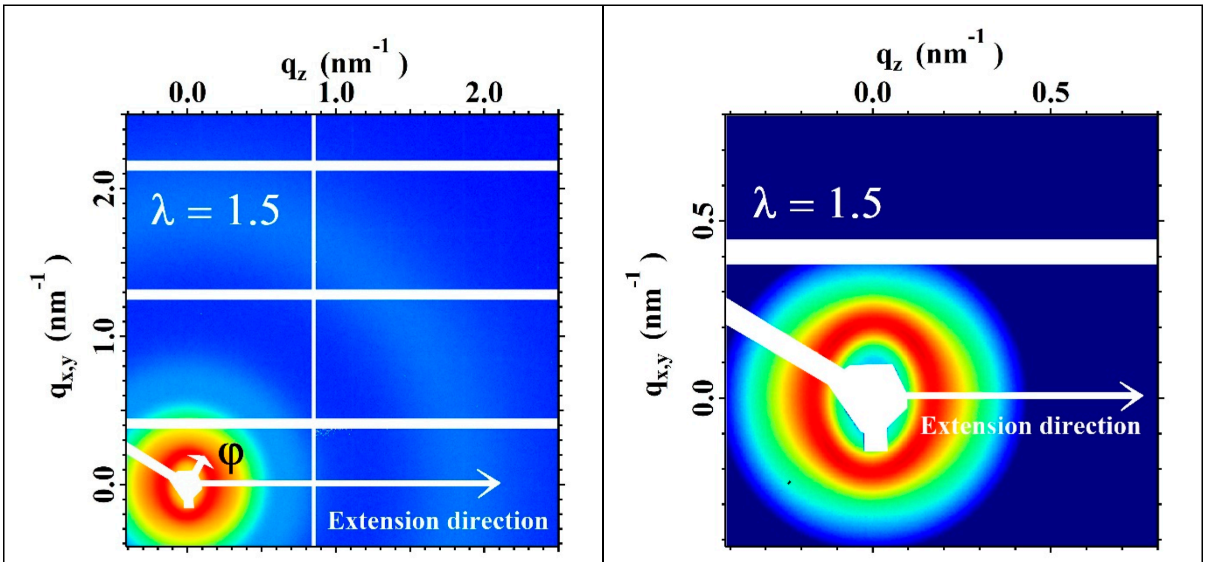

Figure S2. Representative 2D SAXS pattern for sample PDMS_PMMA_2 at λ of 1.5, with the extension direction aligned parallel to the z-axis. The right panel provides a close-up of the central region, highlighting the distinct four-spot pattern.

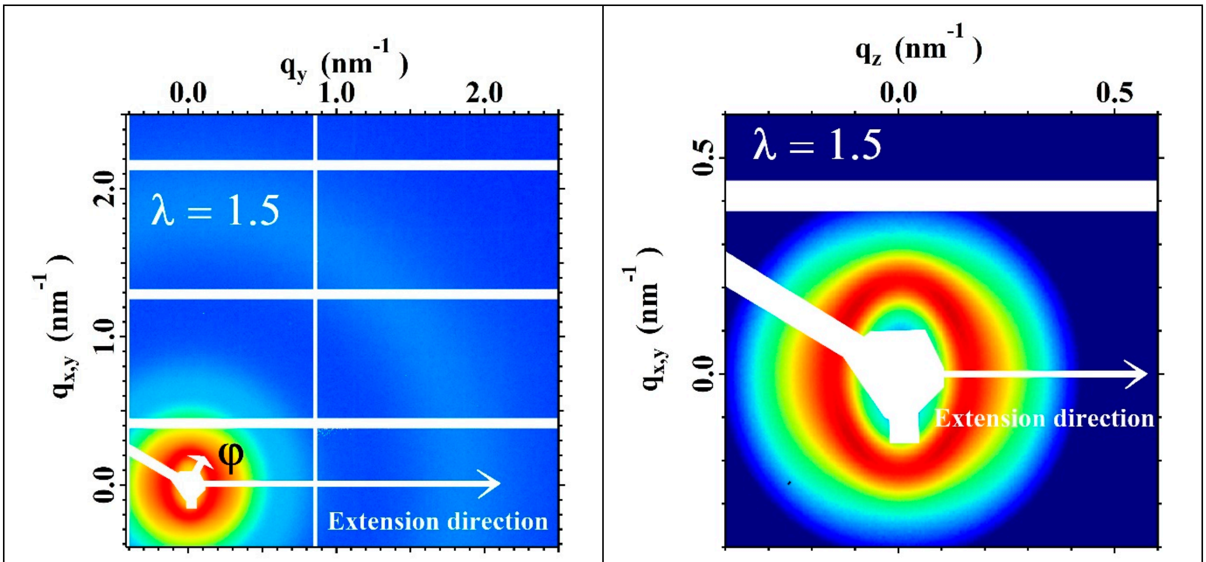

Figure S3. Representative 2D SAXS pattern for sample PDMS_PMMA_4 at λ of 1.5, with the extension direction aligned parallel to the z-axis. The right panel shows the central region, highlighting the distinct four-spot pattern.

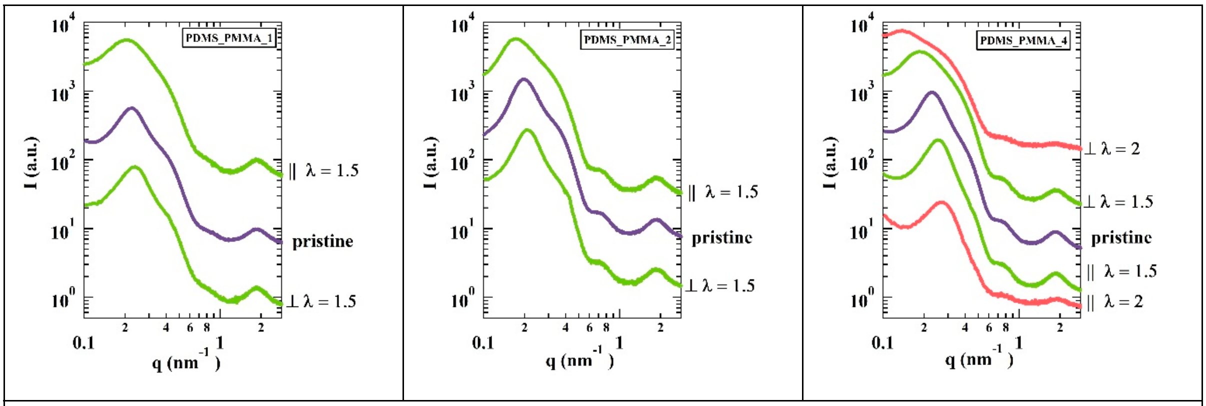

Figure S4. Selected 1D diffraction profiles extracted from 2D SAXS patterns recorded during in-situ stretching experiments on samples PDMS_PMMA_1 (left), PDMS_PMMA_2 (middle), and PDMS_PMMA_4 (right). Profiles are shown for directions both parallel and perpendicular to the stretching direction, with corresponding drawing ratios indicated.

1.2. Poly[nBA-ran-MMA-g-(PIB/PS)] comb-like copolymers

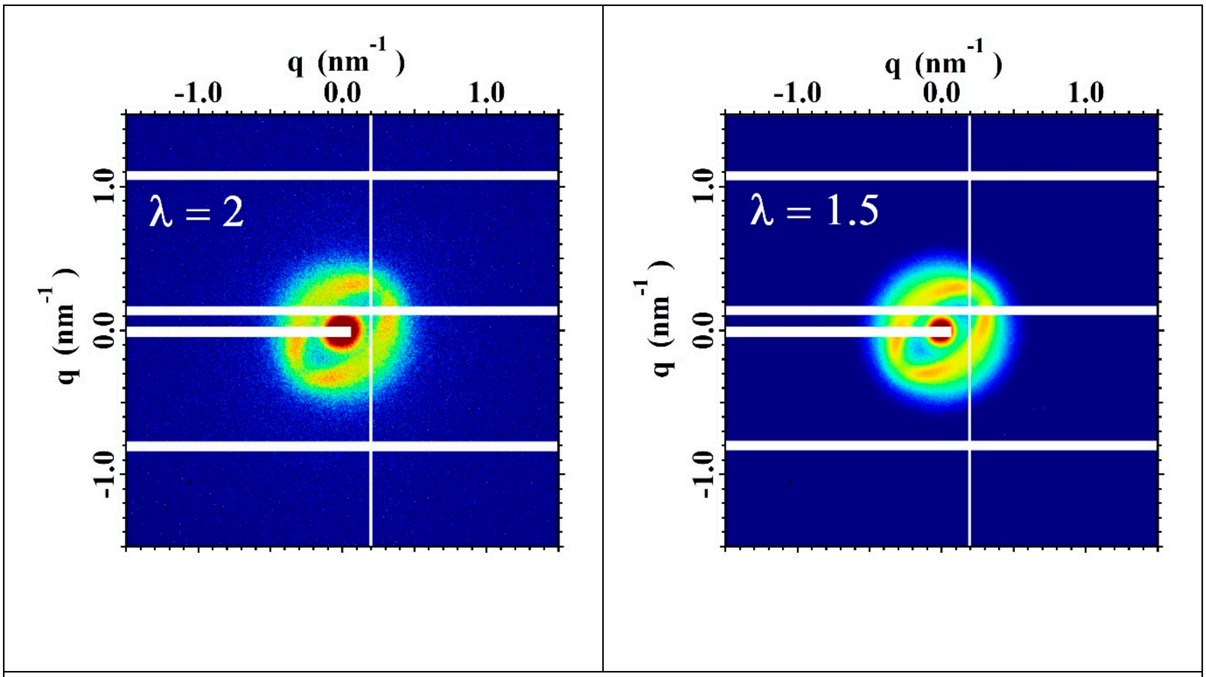

Figure S5. Representative 2D SAXS patterns for samples PIB_PS_1 at λ of 2 (left), and PIB_PS_3 at λ of 1.5 (right), showing the emergence of the characteristic four-spot pattern.

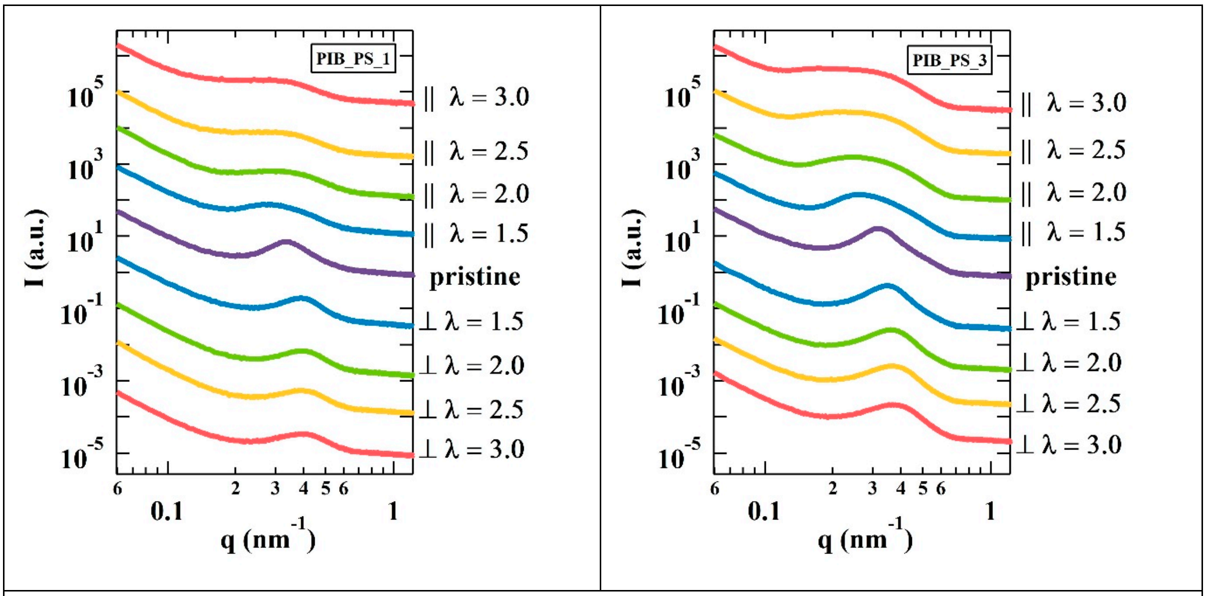

Figure S6. Selected 1D diffraction profiles extracted from 2D SAXS patterns recorded during in-situ stretching experiments on samples PIB_PS_1 (left), PIB_PS_3 (right). Profiles are shown for directions both parallel and perpendicular to the stretching direction, with corresponding drawing ratios indicated.

2. Mechanical measurements

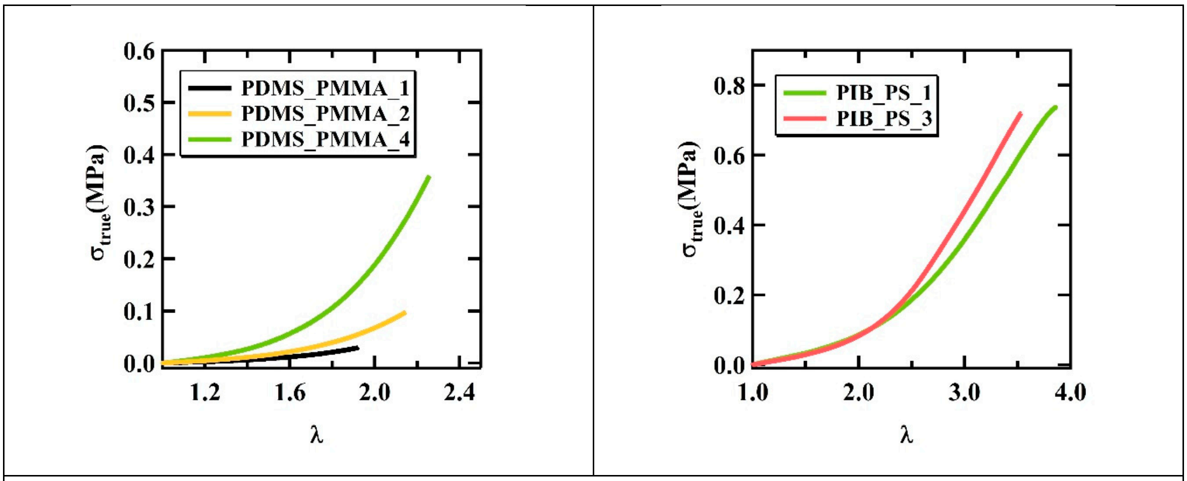

Figure S7. Stress-elongation curves for PDMS_PMMA bottlebrush copolymers (left) and PIB_PS comb-like copolymers (right).

3. Computer Simulations

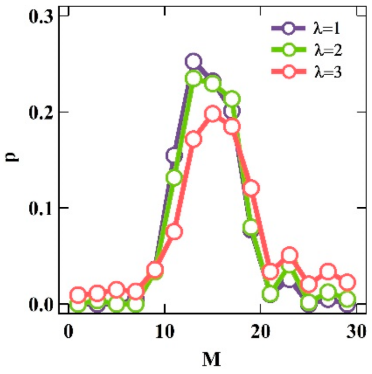

Figure S8. Distributions of aggregation numbers (M) for clusters formed by hydrophobic graft chains at drawing ratios (λ) of 1, 2, and 3, illustrating the evolution of aggregate sizes with increasing deformation. Probability (P) is normalized to unity for each distribution.
